# Conservation and co-option in developmental programmes: the importance of homology relationships

**DOI:** 10.1186/1742-9994-2-15

**Published:** 2005-10-10

**Authors:** Matthias Sanetra, Gerrit Begemann, May-Britt Becker, Axel Meyer

**Affiliations:** 1 Lehrstuhl für Zoologie und Evolutionsbiologie, Fachbereich Biologie, Universität Konstanz, 78457 Konstanz, Germany

## Abstract

One of the surprising insights gained from research in evolutionary developmental biology (evo-devo) is that increasing diversity in body plans and morphology in organisms across animal phyla are not reflected in similarly dramatic changes at the level of gene composition of their genomes. For instance, simplicity at the tissue level of organization often contrasts with a high degree of genetic complexity. Also intriguing is the observation that the coding regions of several genes of invertebrates show high sequence similarity to those in humans. This lack of change (conservation) indicates that evolutionary novelties may arise more frequently through combinatorial processes, such as changes in gene regulation and the recruitment of novel genes into existing regulatory gene networks (co-option), and less often through adaptive evolutionary processes in the coding portions of a gene. As a consequence, it is of great interest to examine whether the widespread conservation of the genetic machinery implies the same developmental function in a last common ancestor, or whether homologous genes acquired new developmental roles in structures of independent phylogenetic origin. To distinguish between these two possibilities one must refer to current concepts of phylogeny reconstruction and carefully investigate homology relationships. Particularly problematic in terms of homology decisions is the use of gene expression patterns of a given structure. In the future, research on more organisms other than the typical model systems will be required since these can provide insights that are not easily obtained from comparisons among only a few distantly related model species.

## Introduction

Evolutionary developmental biology (evo-devo) seeks to unravel the bases of developmental changes in body plan evolution of complex organisms such as animals and plants. The significance of this relatively new discipline is based on the premise that evolution cannot be fully understood without understanding the evolution of developmental programmes [1], and a number of novel conceptual frameworks have emerged from evo-devo research to supplement those of traditional evolutionary biology, such as DEVELOPMENTAL REPROGRAMMING [1-6]. The latter concept describes the process that acts between mutation and selection on the level of the organism, leading from an altered gene product to a new ontogeny and phenotype. Reprogramming has been proposed to constitute an additional evolutionary mechanism because some ontogenetic changes may be promoted by existing developmental mechanisms while other alterations are prevented [1,3,7] (referred to as 'developmental drive' and 'constraint', respectively [8]). It seems therefore likely that evolution can be biased by development, and this may have a powerful impact on the direction of evolutionary change [1,7,8].

During the past two decades it was discovered that most animals, no matter how divergent in form, share specific gene families that regulate major aspects of body patterning, for instance many homeobox-containing genes [9,10], which are even present in the Cnidaria [11]. Recent findings show that morphologically simple organisms often possess genes, such as members of the *pax *gene family, that are homologous and show a high level of sequence similarity to those of higher vertebrates [12-15]. Despite this astonishing extent of evolutionary conservation in developmental regulatory genes across major taxonomic groups, there are also cases where gene expression patterns differ markedly among closely related taxa, for instance in the molecular mechanisms that determine the spatial axes of the tetrapod limb [16]. In the recent past, one of the goals of evo-devo research was to search for putative phylum-specific genes, which may have given rise to phylum-specific evolutionary novelties. However, the view hat new phyla arose in concert with the advent of novel genes has been increasingly challenged [17,18]. Instead, there is mounting evidence that the evolution of lineage-specific body plans does not primarily depend on the invention of new genes but rather on the deployment of new gene regulatory circuitries. Changes in the transcriptional regulation of genes may thus be more significant than changes in gene number or protein function [18,19]. Moreover, the use of 'old' genes for novel structures has recently been demonstrated in a number of instances [20,21].

Among the main controversies that have emerged from evo-devo research is whether or not the utilization of conserved molecular components in developmental programmes across animal phyla can be taken as evidence for a shared developmental function in their latest common ancestor. The alternative would be the co-option of conserved genes and gene pathways to new functions, most likely operating in non-homologous structures. It is therefore fundamental to employ phylogenetic methods and homology criteria meticulously to resolve such issues, including the idea of retention of genetic programmes or 're-awakening' [22-25]. Recent gene expression studies, for instance, have revealed some common molecular aspects of segment ontogenesis between insects and annelids (shared segmental expression of *engrailed *and *wingless*) [26], and between arachnids and chordates (shared role of the notch signalling pathway) [27], which have been used to reinforce the hypothesis that the last common ancestor of all bilaterian animals was segmented [17,28-30]. However, one must be particularly critical about such deep homologies since the probability of gene recruitment to non-homologous roles grows with phylogenetic distance [1,31]. The use of gene expression patterns to establish homologies between morphologically similar features among distantly related organisms is another matter of ongoing debate [1,31,32].

In this review, we provide a brief introduction to evolutionary developmental biology for newcomers to the field who may be overwhelmed by the abundant literature. We outline how recent advances in evo-devo research have changed our understanding of the genesis of species differences and morphological novelties. In particular, we present examples to show that the contribution of phylogenetics to test hypotheses for the interpretation of problems in the evolution of developmental processes [20,33,34] is becoming more and more recognized. We also stress the importance of accurately assessing homology relationships and appropriate phylogenetic sampling of organisms for evo-devo studies.

## Morphological versus Genetic Complexity

It would seem plausible to think that an organism with only a handful of tissue types has a much simpler genetic machinery than morphologically more complex creatures, such as vertebrates. But surprisingly little correlation has been found between genetic complexity and the degree of morphological organization so far. Some recent studies have revealed an astoundingly large number of similarities in the genetic make-up between morphologically complex organisms (e.g., vertebrates) and relatively simple forms (e.g., corals, molluscs). The most spectacular examples come from cnidarians, which are among the most basal metazoan animals composed of only two cell layers and yet exhibit a rather advanced suite of genes, such as *pax, wnt*, and genes involved in organizing the bilaterian head [12,35,36]. About 12 % of the genes found in the EST library of the coral *Acropora *(Anthozoa) were shared with vertebrates but had no match with *Caenorhabditis elegans *or *Drosophila melanogaster *[14]. Until this finding, many of those 'vertebrate genes' were presumed to be lineage-specific, e.g., *Churchill *and *Tumorhead *[14], which are functionally associated with a highly differentiated nervous system. In cases where a particular gene sequence was present in all three animals, the coral sequence matched the human counterpart much more strongly than any of the corresponding *Caenorhabditis *or *Drosophila *sequences [14]. This suggests higher rates of divergence in these invertebrate model systems, and a recent analysis of the Pufferfish genome likewise indicates that many fish proteins have diverged markedly faster than their mammalian homologues [37,38]. Certainly, the genetic complexity of corals is surprising considering that they possess only a few tissue types and a simple nervous system.

**Figure 1:**
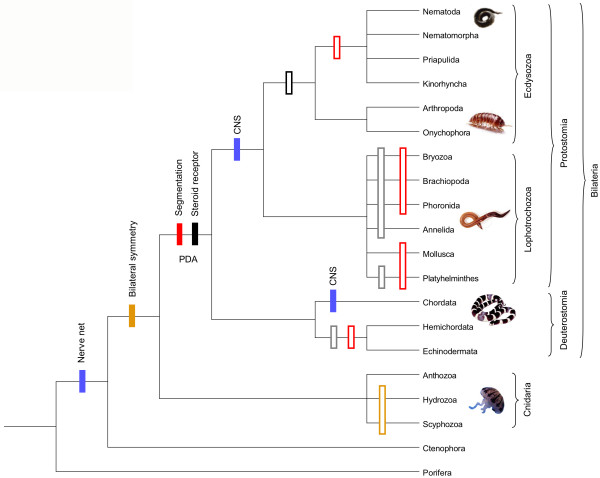
Phylogeny of the Metazoa (adopted and simplified from [112-114]) depicting several new and controversial hypotheses of character evolution with regard to bilateral symmetry, central nervous system, segmentation, and steroid receptors. PDA: last common protostome-deuterostome ancestor; CNS: central nervous system. Filled bars indicate the origin of a character while open bars in the same colour indicate loss of that character. Open grey bars show that data for the assumed loss of steroid receptors are missing.

Similarly, lineage-specific gene losses in some model organisms can obscure ancient orthology relationships among genes. For example, a steroid receptor gene has been discovered in the mollusc *Aplysia*, but no orthologues are known from the fully sequenced genomes of the invertebrates *C. elegans *and *D. melanogaster *[13]. Instead, the sequence was most similar to that of the human estrogen receptor. Much in line with another study [39] these results suggest the unexpected evolution of genes for the major receptor types, such as steroid receptors and thyroid hormone receptors, very early in metazoan history at the base of the Bilateria. Nuclear receptor genes would then be far older than previously thought, with an estimated origin for the protostome-deuterostome split of approximately 960 milllion years ago [40]. Under this hypothesis one has to assume that these genes, previously thought to be vertebrate innovations, were lost independently in several bilaterian lineages (Fig. 1). The most extensive gene losses of ancestral gene families have been reported in *C. elegans *and *D. melanogaster*, with 31.0% and 25.8%, respectively [14,41]. However, the sparse taxon sampling presently hampers a thorough analysis and more informative interpretation of character changes, as does the fact that some critical nodes in metazoan phylogeny are still unresolved [42-44].

The above considerations imply that the ancestral metazoan might have contained many more genes than previously thought (see also [45] on *opsin *genes, and [36] on the *wnt *gene family in sea anemones). The similarity in the genomic repertoire between some invertebrate and human sequences can be explained by the atypical condition (i.e., rapid divergence) of the commonly used model organisms [14,15]. Another important point is whether genes shared between morphologically simple and complex organisms suggest that their ancestor already had the developmental programmes that are now implemented, for example, in modern vertebrates. In several cases, and especially for the corals, it seems more likely that many genes in simple organisms are confined to a single role, probably the ancestral role, while they acquired additional functions later in evolution, which allowed for greater complexity in the organism. Many nuclear receptor genes did probably not yet have their present function in the organisms in which they first occurred [39].

## Evolutionary Genetics of Morphological Novelties

**Figure 2:**
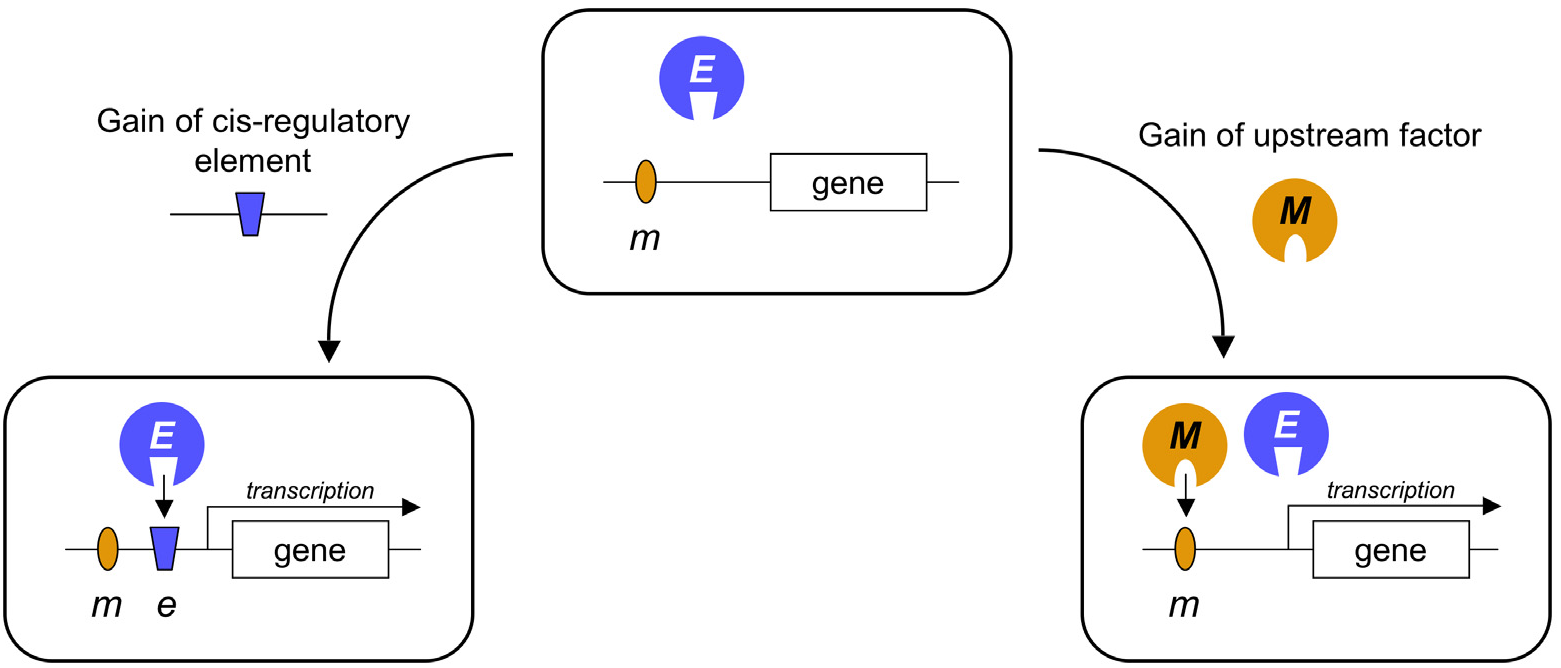
Mechanisms of regulatory evolution. The diagram depicts the two possible modes of regulatory changes that generate novel developmental control mechanisms. New domains of gene activation are achieved by the gain of a novel regulatory element or the gain of an upstream transcription factor (right). If such novel regulatory wiring follows a gene duplication, it may liberate protein evolution and open a developmental trajectory towards co-option (*m/M *= mesodermal enhancer/transcription factor. *e/E *= ectodermal enhancer /transcription factor).

In order to understand how novel characters can arise at the cellular level it is important to study the history of the genes involved and their regulatory interactions. An apparent paradox is that the morphological novelty we observe at the level of different animal phyla is not always reflected in similar changes of gene composition and sequence divergence of the genes controlling development. Instead, the emergence of evolutionary novelties largely appears to be based on the CO-OPTION of already existing genes for new functions. One molecular mechanism acting at the gene level is the acquisition of new regulatory sequences that leads to novel patterns of transcriptional activation [7,22,23,46]. As a consequence, new genes can be recruited into existing regulatory gene networks and result in functional changes to the network. Genes may gain novel expression domains by chance mutations or recombination events in their *cis*-regulatory elements. Alternatively, changes in the expression of upstream transcription factors themselves can initiate activation of target genes in new domains (Fig. 2). When combined with an increase in gene family size through duplications, the acquisition of novel functions in the duplicates (sub- or neo-functionalization) by co-option [47] is a powerful mechanism to increase the pool of developmental building blocks (modules).

The mechanism of co-option is facilitated by the modular character of gene interactions. MODULARITY has become one of the central paradigms of molecular evolutionary biology [48,49]. This concept proposes the use of pre-existing building blocks in novel ways, rather than the origin of completely new elements, as the main source of molecular and regulatory innovations [48,50,51]. In a gene-based, developmental context it suggests that individual genes together perform a given "network function" (e.g., the RTK-Ras or *wnt *pathways) [52]. Such modules can be visualized as being composed of a set of interacting genes that can associate in novel ways with other modules, forming networks of higher level organization. This gene-set, also called the 'GENETIC TOOLKIT', determines the overall body plan and the number, identity and pattern of body parts [53]. It appears that the evolution of metazoan development and body plans is based on an increase in the complexity of the control circuitry regulating an ancestral toolkit of genes, rather than on the invention of novel developmental genes [17]. Extensive comparisons of gene functions in relation to animal evolutionary history will be needed to uncover the ancestral functions of these toolkit genes. Present-day organisms that have retained a particular gene are good candidates for reconstructing ancestral character states if they exhibit shared functions (or consensus functions) [15]. Another possibility to consider is that the ancestral function might have become lost in the course of evolution.

### Proteins required for mineralization were co-opted for vertebrate-specific innovations

**Figure 3:**
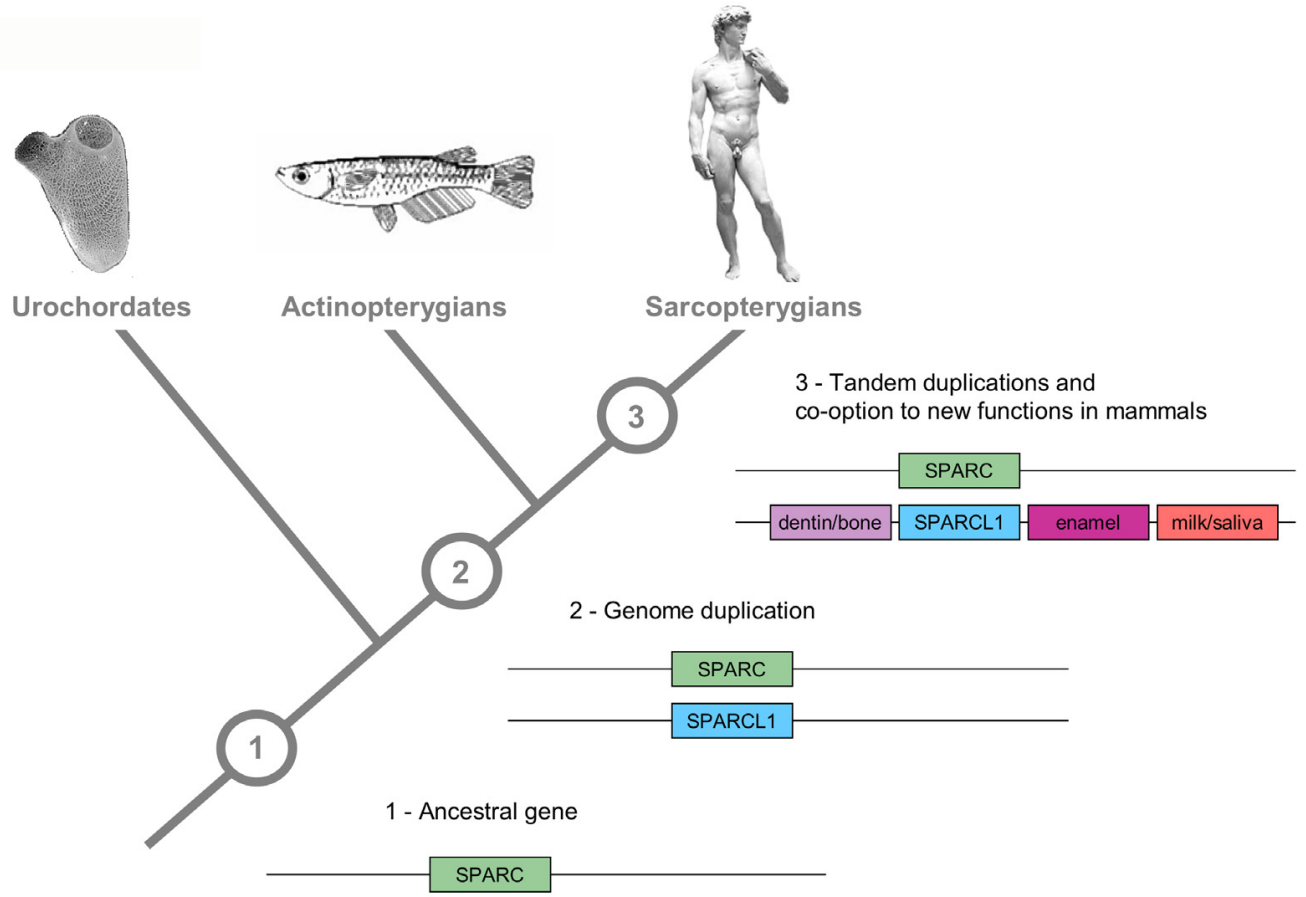
Evolution of the secretory calcium binding phosphoprotein (SCPP) gene family involving gene duplication and co-option. A duplication event of an ancestral SPARC gene (1) in the vertebrate lineage generated two paralogues (2), and one of these (SPARCL1) gave rise to the SCPP gene family through subsequent tandem gene duplications after the divergence of cartilaginous and bony fish (3). In mammals, the formation of tooth and bone tissue is based upon the presence of various members of the SCPP gene family, including genes for enamel SCPPs, dentin/bone SCPPs, as well as milk caseins and salivary proteins. Most of these are closely linked on one chromosome in humans, as shown in the diagram.

One particularly instructive example illustrates this idea. Major transitions in evolution are often accompanied by phylum-specific innovations, such as the occurrence of mineralized tissue in vertebrates [54], which was fundamental to the radiation of modern vertebrates. Its development enabled the evolution of endoskeleton, body armour and teeth, thereby providing adaptive phenotypes for improved locomotion, protection and predation, respectively. At the heart of this tissue type are members of the secretory calcium binding phosphoprotein (SCPP) gene family, which produce the special ionic conditions in the extracellular matrix required for skeletal mineralization. How did this gene family evolve, and what was its likely ancestral function in invertebrates? The SCPP gene family has been traced back to an ancestral gene, SPARC (encoding a non-collagenous bone matrix protein), present in both protostomes and deuterostomes [20]. Tetrapod SCPPs arose from SPARC by several gene duplications followed by co-option for new functions (Fig. 3), and include genes for enamel, dentin/bone, as well as milk caseins and salivary proteins in mammals [55]. Birds have an eggshell SCPP but lack the genes for enamel, milk and salivary-associated proteins, probably reflecting the loss of teeth in birds and the evolution of mammary glands in mammals [56]. SPARC homologues have been identified in a variety of invertebrates where they influence cell behaviour and interactions with the extracellular matrix, rather than being involved in the generation of mineralized tissues. Thus, an evolutionary modification of these proteins seems likely to have occurred through a functional shift from facilitating the stabilization of structural proteins towards enabling more diverse interactions between cells and proteins of the extracellular matrix in the course of vertebrate evolution.

### Regulatory DNA evolution in invertebrates

Significant morphological transformations in the body plan of invertebrates have been found to correlate with developmental changes of *Hox *gene expression patterns [57,58]. Interestingly, as two examples in molluscs demonstrate, some of these changes are not related to the characteristic *Hox *function of establishing pattern along the anteroposterior axis. In the gastropod *Haliotis asinina*, two *Hox *genes (*Has-Hox1 *and *Has-Hox4*) are expressed in the mantle margin, where they have been co-opted into a new developmental role in shell formation [59]. Since morphological novelties derived from the ancestral molluscan body plan are striking in cephalopods, it seems promising to explore the molecular mechanisms of cephalopod innovations. For example, patterns of *Hox *expression in the squid *Euprymna scolopes *strengthen the argument that co-option events are often associated with the origin of new morphological structures [58]. The acquisition of three innovations derived from the ancestral molluscan foot, namely brachial crown, funnel tube and stellate ganglia, could be ascribed to *Hox *gene recruitment during cephalopod evolution. Given the large diversity of molluscan body plans, it has been suggested that this morphological flexibility may result from a relaxation of regulatory constraints on the recruitment of morphological patterning genes [58].

Not surprisingly, variation at the species level is also frequently based upon changes to gene regulation. Expression of the *yellow *gene at the wing tips of the fruitfly *Drosophila biarmipes*, a species closely related to *D. melanogaster*, results in conspicuous black pigment spots. It was shown that, for the evolution of this pigment pattern, the gene's regulatory sequences had gained additional binding sites for highly conserved transcription factors [60]. When experimentally introduced into *D. melanogaster*, these regulatory elements are capable of driving reporter gene expression (and thus *yellow *expression) in the distal-anterior region of the wing. Interestingly, among those newly evolved binding sites is one for the transcription factor *engrailed*, which perfectly illustrates how regulatory pathways already present in the wing have been co-opted to control wing pigmentation through chance mutations of ancestral enhancer sequences. It certainly is an appealing concept that the combinatorial nature of transcriptional regulation creates a large reservoir for morphological diversity, and may provide more variation for natural selection than changes in the gene product alone. Changes in the *cis*-regulatory systems of genes may therefore be more significant than changes in gene number or protein function [19].

### Developmental innovations through protein sequence evolution

Examples in which evolutionary changes in gene regulation lead to morphological changes are numerous [61], yet there are also a number of well studied cases in which changes in protein sequence have been linked to new adaptations (reviewed by [46]). Since many genes have pleiotropic functions, changes to their protein sequence are potentially deleterious. Thus most cases involve gene duplications, as exemplified above for the SPARC gene family, or alternative splicing, in which one copy retains its function while the other acquires a new one. In the latter case, even mutations resulting from detrimental mechanisms, such as frameshift mutations, have been shown to be retained for up to hundreds of millions of years and have evolved new protein functions [62]. Finally, changes to protein-protein interactions can lead to alterations in developmental mechanisms, by integrating novel regulators into existing pathways, or by eliminating old ones. For example, the transcription factor *brachyury *is expressed in the circumference of the blastopore of most animal phyla and its orthologues from most Bilateria are capable of inducing mesoderm when assayed in *Xenopus *animal caps; however, in this assay, *brachyury *orthologues from *Drosophila *and tunicates strongly induce formation of both mesoderm and endoderm, and this is strictly correlated with the loss of a short protein-protein interaction motif, N-terminal of the DNA-binding domain [63]. Interestingly, insects and tunicates have not only lost circumferential blastopore *brachyury *expression independently, but also have a derived mode of gastrulation which is largely independent of *brachyury*. Messenger et al. [64] have recently identified Smadl as the cofactor that binds to the conserved *brachyury *N-terminal peptide and inhibits endoderm induction. The possibility therefore exists that this repression module, which is absent in the diploblastic Hydra, evolved in the bilaterians to separate mesoderm from mesendoderm. In *Drosophila *and tunicates, these tissue types are derived from topologically separate regions, and the derived mode of development may have relaxed the selective pressure required to maintain this motif. It is evident that in the future many more such examples will be found that are associated with the gain or loss of a particular structure or mode of development.

## Phylogeny, Homology, and Gene Expression Patterns

Given sufficient periods of evolutionary time a certain gene or gene cascade may be conserved within a lineage, yet might be highly divergent among lineages. Therefore, while examining the different roles of conserved versus co-opted developmental mechanisms, it is important to recognize that conservation (lack of change) is a relative term whose interpretation depends on the selection of the appropriate phylogenetic framework. Knowledge about the phylogenetic relationships among model organisms and their relatives will thus substantially improve the understanding of developmental processes and uncover general evolutionary patterns [32,65-67]. Phylogenies are statements not only of relationships among taxa, but also about the evolution of characters along the tree. Mapping characters onto a robust phylogeny is a good way to determine if those characters may be homologous [34].

### The basic concepts

**Figure 4:**
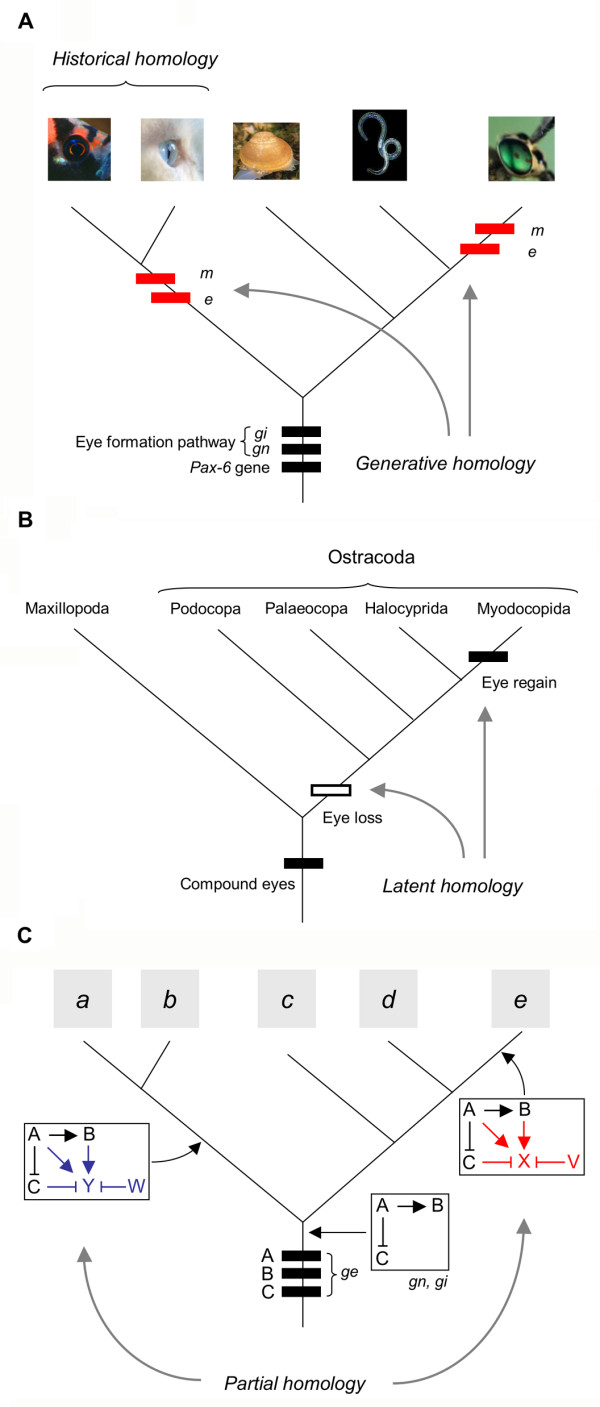
Homology relationships at different hierarchical levels of biological organization (*ge*: gene; *gn*: gene network; *gi*: gene interactions in the network; *e*: embryonic origin; *m*: morphological structure) following the suggestions detailed by Abouheif [65]. (A) Functioning of homologous genes in structures of independent evolutionary origin. In insect and vertebrate eyes, a very similar genetic machinery is used to generate structures that are historically non-homologous and morphologically dissimilar. Note that this generative homology is different from latent homology (B) in that the latter is concerned with morphologically homologous structures. This figure illustrates the difference between two main concepts, historical *vs*. generative homology. (B) Latent homology or re-awakening. If morphologically homologous characters, such as arthropod compound eyes [104], have multiple independent origins on a phylogeny then this may be due to retained genetic programmes that are not expressed in some of the ancestors, but whose functionality has been recovered after further speciation, giving the incorrect impression of convergent evolution. (C) Partial homology of gene networks. Novel genes (V, W, X, Y) are recruited into an ancestral network consisting of genes A, B, and C in two different lineages of taxa (*a*+*b *and *e*). Thus, the black part of the network is homologous (inherited from a common ancestor) while the coloured segments are not.

Homology as a historical concept is typically defined as the shared inheritance of a trait from a most recent common ancestor (e.g., eyes of fish and mammals; Fig. 4A), though plain similarity has inadequately been used in a number of instances (for further discussions see [24,25,68,69]). HISTORICAL HOMOLOGY can be applied to different levels of biological organization, including morphological structures, developmental processes, and genes [23]. Abouheif [70] suggested a hierarchical analysis of homology relationships, which is designed to reveal alternative evolutionary hypotheses, for example the recruitment of homologous genes and networks to function in convergent embryonic and morphological structures (e.g., eyes of vertebrates and insects; Fig 4A). Gene family phylogenies can further be used to indicate if genes are orthologous (arose by speciation and thus are homologous in the strict sense) or paralogous (arose by gene duplication). On the other hand, the concept of GENERATIVE HOMOLOGY (Fig. 4A), or syngeny, uses shared developmental pathways to imply that a given character is generated by the same genetic machinery inherited from a common ancestor [71]. Wilkinson [6] has recently expanded this idea to use a combination of shared key genes (one or more) plus a shared biological or developmental function for which those genes are crucial as an indicator of homology. Both these concepts incorporate the view that the continuity of inheritance of the potential to make a particular trait rather than the continuity of its appearance is what matters most in homologous relationships [72]. The phylogenetic re-appearance of characters (reversal) is often observed even in well established phylogenies, therefore the genetic potential to produce those characters is probably retained, a phenomenon which can be referred to as 'LATENT HOMOLOGY' [69,73] or 'REAWAKENING' [66] (Fig. 4B). Whenever using the term homology it is important to state explicitly which type, subset, or level of homology is alluded to [24,69,74], and to work within a well-defined phylogenetic framework.

**Figure 5:**
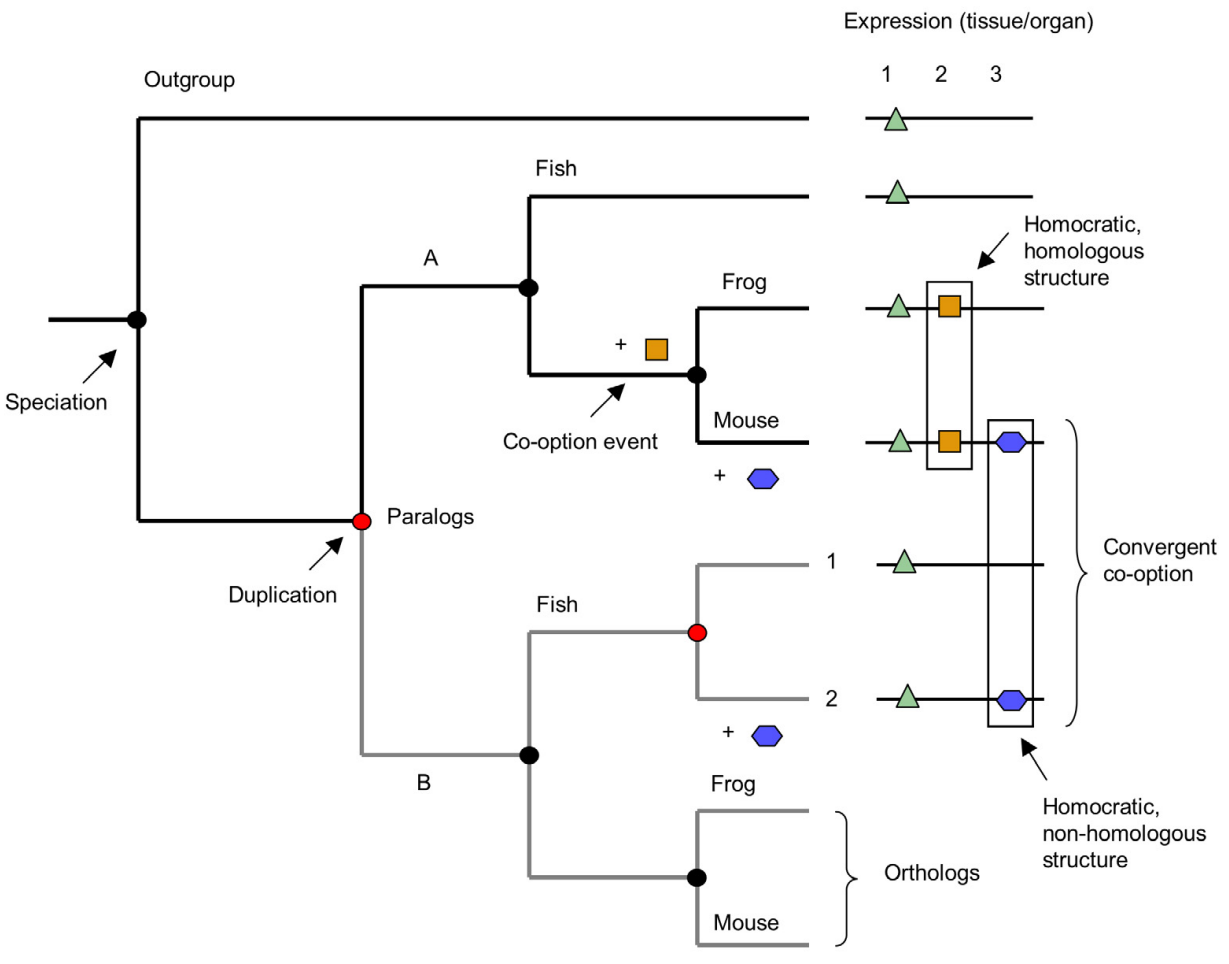
The use of phylogenetic testing in the study of gene expression patterns and to infer ancestral developmental roles. A mixture of orthologous and paralogous genes is included in this phylogenetic tree, therefore reflecting gene relationships rather than relationships of taxa. The phylogeny of taxa can be inferred by analyzing either set of orthologous genes. The two lineages (A and B) of orthologous gene copies are shown in capital letters, and the B lineage is additionally highlighted in grey, while recent paralogues are numbered. Triangles, squares and hexagons indicate gene expression in three tissues/organs, which are grouped by superficial similarity as primary homology hypothesis. Homocratic structures are defined by co-expression of identical patterning genes, but these can be either orthologues or paralogues. In the latter case they do not strengthen a homology hypothesis because convergent evolution is likely (as in the depicted case).

The concept of homology is undoubtedly challenged when it comes to tracking down the evolutionary origins of developmental programmes [24,71,74,75], and the use of gene expression patterns to infer homology is a matter of intense debate [5,23,76]. Of particular difficulty is how to distinguish superficial similarity from phylogenetic information content. As an important rule, comparisons of gene expression patterns to assess homology hypotheses should be restricted to orthologous gene copies, since new expression domains evolving among paralogues are likely to be convergent (Fig. 5). These gene relationships may be complex and thus need to be tested based on a phylogenetic tree including the whole gene family by identification of the timing of speciation events relative to gene duplications [23] (Fig. 5). An analysis of the *Snail/Slug *gene family revealed functional modifications after the original copy had become duplicated during vertebrate evolution. In addition, the study demonstrated unexpected evolutionary changes in *Snail/Slug *expression domains, which imply a large degree of plasticity and highlight the risk of using expression or function as homology indicators when studying the evolution of gene families [32]. Patterns of gene expression are particularly complicated to interpret in terms of homology because they interconnect different levels of biological organization, in this case genes and morphological structures [23]. Conserved expression patterns do not necessarily indicate conservation of gene function, and we know today that homologous genes can be expressed in structures that have different evolutionary origin (thus are non-homologous), such as *distal-less *in animal appendages [76] (see also previous sections for more examples). Likewise, similar developmental roles may be the result of convergent evolution [25]. To overcome some of these difficulties and ambiguities in terminology, the term HOMOCRACY was recently proposed to describe organs/structures that are governed by identical patterning genes [76]. It is important to note that a given structure can be both homocratic and homologous, thus the two terms are not mutually exclusive (Fig. 5).

### Gene network evolution

Behind the scenes are complex interacting gene networks (pathways) that form the genetic machinery required for the origin and functioning of morphological structures. These networks can be considered a distinct level of biological organization, the homology relationships of which may differ from other such levels [22,70]. However, some authors have dismissed the strictly hierarchical view of biology calling for a combinatorial approach to homology [74,77], because interactive combinatorial processes, such as co-option and modularity, play a significant role in biological systems. One consequence of this would be to accept that homology assessments cannot be reduced to a yes-or-no question even at the molecular level [74,78]. The obvious problem is how much (and which parts) of a given entity, for instance a gene network, must be continuous between lineages. Clearly, two networks are homologous if all genes and their interactions are derived from an identical network in the most recent common ancestor. Quite often networks share certain elements but differ in others, and thus they are neither fully homologous nor independently evolved; rather, they might be considered partially homologous. The recruitment of novel genes into an existing regulatory gene network, for instance, will lead to partial homology (Fig. 4C). As a consequence, gene expression patterns need to be evaluated at the level of gene networks to reveal their true evolutionary relationships [70,79]. Studying closely related species in a phylogenetic context is helpful in this case, because smaller changes are expected, and this knowledge can then be used to connect more ancient character states. For example, the wing patterning network in *Drosophila *is well studied, and when the expression patterns of its constituent genes in wingless castes of ants were compared, the results provided surprising insights into the evolution of wing polyphenism: several closely related species do not share a common mechanism to interrupt wing development in the wingless castes, which is an unexpected finding given that a common origin of all wingless castes can be assumed [33].

### Similar expression patterns do not necessarily indicate homology

We have emphasized along with other authors [5,73,76] that gene expression patterns should not be used to infer morphological homology of structures without employing phylogenetic criteria to test hypotheses about orthology of genes as well as partial homology, or convergence of gene networks. In addition, genes that are expressed during basic cellular processes, such as cell proliferation or epithelial-mesenchymal transitions, are likely to be frequently utilized in non-homologous organs. As they have a high developmental and evolutionary constraint, they are not informative to support homology of structures. Conversely, if homology of structures is to be based upon gene expression, members of the genetic toolkit should be consulted, as they control diverse and general patterning processes. Some recent studies that seem to indicate deep homologies in the body plan of animal phyla, although employing this latter strategy, should nevertheless be interpreted with caution. For example, Lowe et al. [80] proposed that a comparable expression pattern in the nervous system of chordates, hemichordates and *Drosophila *is an indication of homology (at least 14 out of 22 genes involved in neural patterning), suggesting that the ancestor of deuterostomes (and probably all bilaterians) had a diffuse nervous system that was centralized independently in arthropods and chordates (Fig. 1). This idea disagrees with the prevailing theory of a single origin of the central nervous system with a dorsoventral axis inversion, which is supported by the inversion of TGFβ-signalling in chordates and *Drosophila *[81]. The example further illustrates the undesirable fact that there is no obvious boundary as to which number of genes need to be co-expressed to proclaim a structure homologous, i.e., would 10 out of 22 genes still justify homology of these nervous systems? As with morphological analyses, similarity measures cannot be used as evidence to indicate common descent, and are thus incongruent with the concept of homology. Such approaches are clearly phenetic and by no means phylogenetic. In another example, the use of homologous genes to achieve bilateral symmetry in larvae of sea anemones was used to infer that bilaterality originated before the split of Cnidaria and Bilateria [82] (Fig. 1). But is bilateral symmetry of sea anemones really homologous to that observed in more derived animals or did it arise by convergent evolution [83]? To answer this question we concur with Holland [31] that if orthologous genes are used to control the development of a similar structure in two otherwise morphologically different animals, this lends some support to the hypothesis of homology but is in itself not sufficient and requires more rigorous tests. Further, early metazoan interrelationships have remained particularly difficult to understand, once more stressing the importance of phylogenetic certainty in relation to the co-option or homology question.

Nevertheless, one adequate method of testing morphological homology hypotheses by using gene expression data has recently been proposed [84]. This approach involves the investigation of changes in developmental systems in a parsimony analyses by mapping gene expression data onto a phylogenetic tree along with other characters. Different homology hypotheses can then be evaluated in terms of the number of evolutionary steps required for each hypothesis to be valid, and the most parsimonious solution (involving the smallest number of steps) can be identified. An example can be seen in Figure 5, depicting a single co-option event leading to shared gene expression in organ 2 in frog and mouse. This supports the homology of organ 2 in these two species. But if instead we were to assume organ 2 in frog as being homologous to organ 3 in mouse, then two independent changes in gene expression would be required (gain of the purple hexagon and loss of the orange square in organ 3 to explain the observed gene expression pattern), which would be less parsimonious and thus would not support this homology relationship. In this way, several competing homology hypotheses can be compared in a combined analysis including many genes and other characters [84]. In general, whenever a co-option event can be indentified as a SYNAPOMORPHY for a set of taxa, it should be informative to support the homology of a structure. Gene expression data could also be useful to distinguish between convergence and re-awakening, as in the former case we expect distinguishing features to reflect different origins of two independently evolved structures while in the latter we do not (see also chapter on the retention of genetic programmes).

## What does Shared Genetic Potential suggest: Conserved Developmental Programmes or Repeated Evolution?

Despite growing evidence for a widespread conservation of the genetic toolkit that is used to produce the complex body plan of bilaterian animals, there is reason to believe that the last common ancestor (Urbilateria) did not necessarily employ the same developmental programmes of extant animals. One principal idea is that the function of many homologous developmental genes in a last common ancestor was of the same general kind as now observed, but in a different developmental context [4]. Conserved genes, or entire networks (modules), might have been co-opted repeatedly into new regulatory regions or morphological structures. If the available genetic toolkit is of limited size, then the possibility of co-option for similar functions, i.e., the repeated evolution of analogous developmental processes, does not appear unlikely [76]. For instance, the homeobox gene *distal-less *(*dll*) and its vertebrate homologue *dlx *are expressed across extant animals in various types of appendages (e.g., vertebrate limbs and echinoderm tube feet) that are clearly non-homologous [22,85]. Shared functions of *dll *genes among animal phyla are few and very general, so that the consensus function is reduced to a general role in regulating cell proliferation [76]. Hence, the bilaterian ancestor probably had no legs but perhaps some inconspicuous body wall outgrowths triggered by *dll *expression. Co-option of this pre-existing mechanism into more specific building blocks (e.g., for structures that grow out distally but are historically non-homologous), could have subsequently occurred during the evolution of different animal phyla.

It is currently intensely debated whether segmentation in different animal phyla has had a common origin or not [28,29,85-88]. Specifically, the question is whether the last common protostome-deuterostome ancestor was already segmented or whether segmentation arose on three separate occasions in arthropods, annelids and vertebrates (Fig. 1). Current views on this have partly changed towards the 'single origin of segmentation' hypothesis due to the finding that *notch *and *delta *genes participate in the segmentation of both spiders and vertebrates [26]. By contrast, segmentation in *Drosophila *is *notch *and *delta *independent. The more basal phylogenetic position of spiders in relation to *Drosophila *suggests a derived mode of segmentation in the latter [34], and thus allows the evaluation of more distant ancestors within the arthropod clade. Similarly, the arthropod-like expression pattern of *engrailed *and *wingless *genes in segment formation of the annelid *Platynereis *points to a segmented ancestor of all protostomes [27]. However, there are also reasonable objections to the 'single origin of segmentation' hypothesis (e.g., for parsimony reasons [89]). Part of the problem might reside in the definition of segmentation since some authors relax the definition to include other repeated structures, such as paired coeloms in echinoderms, which would render the lack of segmentation an uncommon trait throughout the animal kingdom [29,34]. Furthermore, segmentation is a mesodermal process in annelids and vertebrates, whereas in arthropods it is primarily ectodermal [88]. It is therefore conceivable that the same deeply conserved modules have been co-opted for similar functions many times, giving rise not only to the morphologically quite different types of animal segmentation but also to segmented tissues of different embryonic origin found among metazoans. Segmentation would then be a case of repeated evolution not implying the existence of a segmented Urbilateria.

The way in which *Pax6 *and its associated genes are involved in eye development across the Metazoa suggests a shared genetic potential for the occurrence of eyes [90]. Yet the phylogenetic pattern of the distribution of eye structures (adopting the concept that a simple photoreceptor is not an image-forming eye [91]) is clearly polyphyletic, which indicates multiple independent origins from forms lacking eye development [91,92] (Fig 4A). The current situation most likely reflects successive losses and gains of the use of the *Pax6 *network during the evolution of metazoan animals [6]. Some authors have suggested that *Pax6 *has become integrated into several independently evolved genetic programmes to regulate particular aspects of eye development [76,93,94], rather than being a master regulator of eye development [95]. Therefore, repeated utilization of similar genetic pathways involving pre-existing building blocks may emerge as a common theme in animal evolutionary history. Completely new morphological structures can likewise evolve by the integration of independent anatomical entities, e.g., cell populations, which differ in their structure and tissue of origin. According to recent evidence the vertebrate eye is a compound structure comprising two types of light-sensitive cells (rhabdomeric and ciliary receptors) with independent evolutionary histories [45]. The presence of ciliary photoreceptors containing an opsin similar to those of vertebrates in the brain of the ragworm *Platynereis *(Annelida) suggests that both receptor types were present in Urbilateria. Thus, it is straightforward to propose that vertebrate and invertebrate eyes are partially homologous since they contain homologous (e.g., rhabdomeric photoreceptors) and non-homologous cell types derived from different germ layers.

## Testing Evolutionary Hypotheses

The study of character evolution has become one of the central aspects in modern phylogenetic analysis due to its power to reveal and test different evolutionary hypotheses [96,97]. One of the essential prerequisites of this approach, however, is that for those tests to be valid one needs to work within a highly resolved and robust phylogenetic framework. Not only can simple character changes and homology relationships be investigated but also the existence of controversial evolutionary mechanisms. This is particularly useful for assessing conceptual problems in evolutionary developmental biology.

### Developmental bias

**Figure 6:**
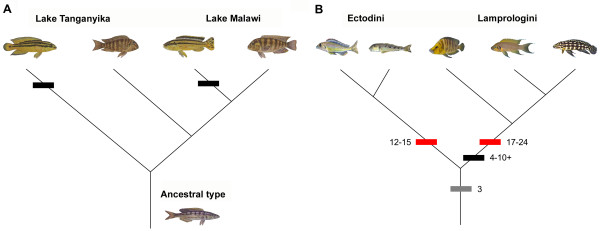
Phylogenetic character distributions that would support developmental bias. (A) Convergence – If certain characters appear repeatedly in a phylogeny despite the obvious absence of exogenous selective causes, this may suggest an evolution biased by development. A possible example are the horizontal bar patterns evolved in parallel in Lake Tanganyika and Lake Malawi cichlids (indicated by black hashmarks), for which similar developmental mechanisms might be used. (B) Phylogenetic inertia (or constraints) – If characters show significant resistance against evolutionary change despite substantial environmental heterogeneity, then at least part of these constraints may be developmentally induced. Different species-rich tribes of East African cichlids show a relatively conservative number of spines of the dorsal fin (Ectodini: 12–15 with the exception of *Enantiopus *having 16–19; Lamprologini: 17–24, indicated by red hashmarks) and of the anal fin (Ectodini and most other cichlids: 3; Lamprologini: 4 or more [98], indicated by grey and black hashmarks) despite having evolved manifold body shapes.

It has been suggested that evolution can be biased by development in that, starting with a particular ontogeny, some phenotypes might be easily produced while others are unlikely or even impossible [1,7]. This idea is also perfectly compatible with the parsimony principle, since an already existing structure will more likely be used for a new purpose rather than a whole new structure will evolve for the same purpose, i.e., instead of evolving gills whales adapted the respiratory mechanisms of their lungs [8]. Thus, it appears that mutation and selection are not the only forces that have shaped the 'possible creatures' inhabiting this planet. In order to assess the significance of DEVELOPMENTAL BIAS  as an evolutionary mechanism one can devise hypothetical character distributions on a phylogeny that would support this notion. Two main categories of character evolution in which developmental bias could have played a major role can be perceived (Fig 6.). Firstly, the convergent evolution of a character towards a similar morphology, such as colour patterns in Lake Tanganyika and Lake Malawi cichlids, may be indicative of biased evolution or selection (Fig. 6A). Secondly, a tree topology that suggests phylogenetic constraints (also known as PHYLOGENETIC INERTIA) may imply that at least part of this pattern may be due to developmental constraints. For instance, several tribes of cichlids often show relatively constant ranges in the number of spines of their dorsal and anal fins [98] (Fig. 6B). In general, phylogenetic constraints refer to taxon-specific limitations forcing a taxon into certain combinations of characters regardless of where that taxon occurs, i.e., an explicitly non-adaptive interpretation [99,100]. There are several methods available to test for phylogenetic inertia, such as autocorrelational analysis and phylogenetic correlograms [100,101].

Once a phylogenetic pattern of character distribution that is suggestive of biased evolution has been established, the problem arises how to distinguish developmental bias from highly similar selection pressures. This is by no means an easy task and will certainly present a great challenge for the future. One possible test for this could be to rule out adaptive explanations for certain morphologies by employing character correlation analysis with environmental variables and to estimate fitness values to test for actual selection. Another approach could be based on the assumption that the likelihood of bias increases with the number of species showing that particular morphology in relation to evolutionary distance. Though theoretically well explored [8], available evidence for developmental bias is presently scarce, and probably the most convincing example is that of the 3,000 and more species of centipedes all have odd numbers of leg-bearing segments. To invoke selection for this phenomenon seems unreasonable [102]. Further, floral symmetry patterns in angiosperms have been proposed as an example for bias-led evolution [103].

### Retention of genetic programmes

In evolution, certain structures may be lost and later re-appear yet the genetic potential to produce those structures may be retained, even though the structures are not continuously present in all ancestors (Fig 4B). This observation has become known as latent homology or re-awakening [66,69,73]. Re-awakening may represent a valid hypothesis in cases where phylogenetic character distributions suggest the reappearance of a character that would be considered homologous on morphological grounds. Many instances of reversals that appear in robust phylogenies might actually be hidden cases of latent homology [69]. There are, however, only a few not extremely well-supported examples of this phenomenon but methods could be established to test for this interesting case of silencing and re-expressing of genetic pathways more meticulously. If dormant genes can be re-activated after further speciation, we expect high similarities in the developmental systems between the lost and the regained structure, i.e., these structures should be generatively homologous. The most convincing way of testing for re-awakening after having established a reliable character evolution would be to demonstrate the functionality of the genetic programme by experimental induction of the putatively re-evolved trait in the species that do not exhibit the trait.

Several species in the genus *Xiphophorus *display a so-called sword, which is a sexually selected extension of the ventral tail fin. The molecular phylogeny shows that the sword was lost once and later re-evolved at least twice in different branches, which suggests re-awakening of the 'sword-developing' programme [66]. Further, swords could be induced in some of these naturally sword-less species through testosterone treatment, thereby making a much stronger case for this hypothesis. It should be noted, however, that swords could also be induced by artificial selection in more distantly related species and that the sword as a morphological structure is rather loosely defined.

In arthropods, there is molecular phylogenetic evidence for the independent origin of compound eyes (Fig. 4B). Myocopids are the only group within the Ostracoda (Crustacea) that have compound eyes, and these are nested phylogenetically within several groups that lack this kind of eye [104]. Maximum likelihood methods of ancestral-state reconstruction were highly significant in supporting the independent origin of compound eyes from eyeless ancestors. However, the ommatidia of many diverse groups of arthropods (including the Maxillopoda as outgroup to Ostracoda) have an arrangement of photoreceptive cells different from the regained eyes in ostracods, which casts some doubt on their homology from a morphological point of view. It will be interesting to know whether the same genetic pathways are used to produce these slightly different types of compound eyes, i.e., if they are generatively homologous, which would strengthen the case for re-awakening.

A recent example from stick insects suggests that wings have re-evolved as many as four times during the radiation of this group [105]. An alternative interpretation would involve 13 independent occasions of wing loss, which is the less parsimonious solution (requiring more evolutionary changes). But accepting the alternative hypothesis would not appear implausible if a different method of CHARACTER OPTIMIZATION were used, i.e., one that assumes loss to be more likely than re-appearance [34]. This example shows that care has to be taken while establishing the basic requirement for re-awakening, which is a robust phylogenetic hypothesis of character evolution. In addition, phylogenies based on morphological characters will be affected in a negative way by re-awakening because the reappearing character causes homoplasy and will be incongruent with other characters. Although re-appearance of a character using the same genetic machinery is evolutionarily truly parsimonious, conflicting hypotheses may arise when applying the parsimony principle for phylogenetic tree construction in such cases.

### The importance of phylogenetic taxon sampling

To determine the mode and direction of character evolution in a phylogeny most commonly the outgroup comparison is used [96,97]. The outgroup constitutes a species set that is as closely related to the ingroup as possible but must not be part of the ingroup. Shared character states between ingroup and outgroup indicate the ancestral state of a character, for example using frogs as outgroup to reptiles shows that having four legs is an ancestral trait. Very often only a single species is used as outgroup, which can be misleading if this particular species is not representative for the whole group – it is obvious that caecilian amphibians would not be good candidates to infer ancestral character states for limb development. In order to draw firm conclusions on ancestral character states extensive taxon sampling should be performed so that ingroup and outgroup contain a broad range of evolutionarily informative (i.e., phenotypically diverse) species. As a consequence, reliably defining ancestral character states in a phylogeny is impossible without the aid of non-model organisms since the typical model systems are often not characteristic for an entire clade, i.e., often show a high proportion of advanced character states. A good example for this comes from the *Hox *genes of *C. elegans *[106], which, as a member of the ecdysozoan clade, was expected to have a relatively large number of these genes. However, the investigation of a representative range of taxa shows a prominent loss of *Hox *genes during nematode evolution in which the most derived state (at least five *Hox *genes fewer than most other Ecdysozoa) was observed in the model *C. elegans*.

The attempt to reconstruct more gradual sequences of change emerges as a general requirement to deduce reliable conclusions on evolutionary events. This is also the case when studying the relationship of molecular changes and phenotypic evolution. *Hox *gene expression in crustaceans was found to be tightly correlated with alterations in morphology in that a shift in the anterior-posterior border of *Ubx *expression coincides with the occurrence of feeding appendages (maxillipeds). The latter could be demonstrated by investigating different evolutionary stages represented by seven genera from six crustacean orders [57]. Without sampling those intermediates, which often are "minor" taxa, false correlations between molecular and morphological evolution are expected because multiple character changes will be compressed into a single event on a phylogenetic tree [34]. This highlights the importance of reaching confidence on ancestral nodes as the crucial point in interpreting character evolution.

## Conclusion

Understanding the high degree of conservation in genes and gene cascades on one hand, and the large morphological diversity on the other will be a great challenge. Molecular evolutionary analyses will have to focus more on the interaction context of gene networks and the concept of modularity rather than on individual genes [49,107]. It also emphasizes the need for correctly assessing the degree of homology *vs*. homoplasy in defining common components of developmental pathways. For many questions in comparative biology, and also in evo-devo research, the availability of a well-supported phylogeny among the study organisms is of paramount importance. Phylogenomic approaches (using multiple gene loci sequence data) to display evolutionary relationships among model organisms will certainly appear more frequently [43,108]. One obvious drawback of these studies is that data are available for only a small number of taxonomic representatives. In many fields of research, especially in medical applications, conclusions still tend to be drawn from a small number of model systems, though there is clear evidence that integration of many species sheds new light onto old questions. The Cnidaria and Porifera are good candidates in the hunt for new genetic inventions [14,36,109], as these two phyla are basal to the Bilaterians. Sponges (Porifera) possess the most basic features of the metazoan bodyplan. Thus, comparative genomics including this animal group emerge as a promising tool to gain insights into the genetic architecture of the hypothetical Urmetazoa as the earliest common ancestor of all metazoans [110,111]. Further studies in this direction will finally be able to reveal the minimal toolkit assembly for metazoan animals, and open up new research avenues in the evo-devo field.

## Authors' contributions

MS and M-BB carried out most of the initial literature search and drafted the first version of the manuscript. GB expanded the review and, together with MS, wrote the final version. AM revised the manuscript critically, which was read and approved by all authors.

## Glossary

### CHARACTER OPTIMIZATION

The most parsimonious reconstruction of the states of a character (e.g. 'winged' and 'wingless') when mapping that character onto a pre-established (usually molecular) phylogeny.

### CO-OPTION

An evolutionary process in which existing features become adapted for new functions, e.g., the change of gene function to new pattern forming processes.

### DEVELOPMENTAL REPROGRAMMING

Modification of the prevailing ontogenetic trajectory within an evolutionary lineage.

### DEVELOPMENTAL BIAS

The concept that evolution may be biased by development because some ontogenetic changes may be more likely than others, which is also termed 'developmental drive'. The unlikely or impossible changes are referred to as 'developmental constraints'.

### GENERATIVE HOMOLOGY

Generation of a character by employing shared genetic mechanisms or pathways that consist of a set of homologous genes, while the character itself need not be homologous on morphological grounds.

### GENETIC TOOLKIT

The toolkit is composed of a small fraction of all genes that are widely conserved among different animal phyla, and which generally control the expression of other genes.

### HISTORICAL HOMOLOGY

Biological structures or traits are homologous if they were inherited from a most recent common ancestor, which can be evaluated on a phylogenetic tree.

### HOMOCRACY

Organs or structures characterized through the expression of functionally identical patterning genes but the homology relationships of these genes may be unresolved (and thus may include orthologues and paralogues).

### LATENT HOMOLOGY OR RE-AWAKENING

Phylogenetic re-appearance of a morphologically very similar character (being homologous in the sense of the homology criteria of position and special qualities [69]) for which the genetic potential to produce that character is retained.

### MODULARITY

Biological level of organization into a set of interconnected units in an organism.

### PHYLOGENETIC INERTIA

The tendency of traits to resist evolutionary change despite environmental perturbations.

### SYNAPOMORPHY

A shared derived character state that is indicative of a phylogenetic relationship among two or more taxa.
